# Introgression of Herbicide-Resistant Gene from Genetically Modified *Brassica napus* L. to *Brassica rapa* through Backcrossing

**DOI:** 10.3390/plants13202863

**Published:** 2024-10-13

**Authors:** Subramani Pandian, Young-Sun Ban, Eun-Kyoung Shin, Senthil Kumar Thamilarasan, Muthusamy Muthusamy, Young-Ju Oh, Ho-Keun An, Soo-In Sohn

**Affiliations:** 1 Department of Agricultural Biotechnology, National Institute of Agricultural Sciences, Rural Development Administration, Jeonju 54874, Republic of Korea; pandiannsp7@gmail.com (S.P.); novis7@naver.com (Y.-S.B.); novis7@korea.kr (E.-K.S.); seninfobio@gmail.com (S.K.T.); biotech.muthu@gmail.com (M.M.); hogni7873@korea.kr (H.-K.A.); 2 Institute for Future Environmental Ecology Co., Ltd., Jeonju 54883, Republic of Korea; 50joo@hanmail.net

**Keywords:** *Brassica napus*, *Brassica rapa*, interspecific hybridization, herbicide resistance gene, simple sequence repeats, backcrossing

## Abstract

Interspecific hybridization between two different Brassicaceae species, namely *Brassica rapa* ssp. *pekinensis* (♀) (AA, 2n = 2x = 20) and genetically modified *Brassica napus* (♂) (AACC, 2n = 4x = 38), was performed to study the transmission of a herbicide resistance gene from a tetraploid to a diploid *Brassica* species. Initially, four different GM *B. napus* lines were used for hybridization with *B. rapa* via hand pollination. Among the F1 hybrids, the cross involving the *B. rapa* (♀) × GM *B. napus* (♂) TG#39 line exhibited the highest recorded crossability index of 14.7 ± 5.7. However, subsequent backcross progenies (BC1, BC2, and BC3) displayed notably lower crossability indices. The F1 plants displayed morphological characteristics more aligned with the male parent *B. napus*, with significant segregation observed in the BC1 generation upon backcrossing with the recurrent parent *B. rapa*. By the BC2 and BC3 generations, the progeny stabilized, manifesting traits from both parents to varying degrees. Cytogenetic analysis revealed a substantial reduction in chromosome numbers, particularly in backcrossing progenies. BC1 plants typically exhibited 21–25 chromosomes, while BC2 progenies showed 21–22 chromosomes, and by the BC3 generation, stability was achieved with an average of 20 chromosomes. SSR marker analysis confirmed the progressive reduction of C-genome regions, retaining minimal C-genome-specific bands throughout successive backcrossing. Despite the extensive elimination of C-genome-specific genomic regions, the glyphosate resistance gene from the male parent *B. napus* was introgressed into BC3 progenies, suggesting that the glyphosate resistance gene located and introgressed in A-chromosome/genome regions of the Brassica plants.

## 1. Introduction

Canola (*Brassica napus* L.) (AACC, 2n = 38), which is also known as oilseed rape, is a vital global oil-producing crop [1]. This amphidiploid species resulted from the hybridization of *Brassica rapa* (A genome, n = 10) and *Brassica oleracea* (C genome, n = 9) [2]. *B. napus* became an agricultural crop relatively recently, around 7500 years ago [3]. It displays various growth habits, including spring, winter, and semi-winter varieties, though genetic diversity within a specific growth habit type is quite limited [4]. *B. rapa* is a diverse species with subspecies ranging from oilseed varieties to leafy vegetables, and some of these subspecies are commonly found in the wild near waterways or as agricultural weeds [5]. With the increasing commercialization of genetically modified (GM) crops, *B. napus* has been engineered to include important genes with various functions. Most commercially used GM *B. napus* varieties carry transgenes conferring resistance to herbicides like glyphosate, glufosinate, and bromoxynil [6,7]. The specific effects of these herbicides promote the cultivation of GM canola, raising the risk of herbicide-resistant genes spreading through hybridization with closely related species [8].

Interspecific hybridization between domesticated crops and their wild counterparts facilitates the exchange of genes across species, promoting the gradual spread and combination of traits [9,10]. Concerns about gene flow are particularly significant when evaluating the environmental risks associated with cultivating or importing genetically modified (GM) crops in countries that prohibit GM crop cultivation [10,11]. Unlike crops such as maize, potato, or cotton, which rarely form feral populations or interbreed with closely related species, the potential for gene flow from cultivated oilseed rape (*B. napus*) to its wild relative, *B. rapa*, is substantial [12]. This gene transfer can occur not only within *B. napus* fields but also in naturalized populations where both species coexist, including roadsides, agricultural areas, transportation routes, ports, and mixed-crop environments [10,13]. The transfer of transgenes, particularly herbicide-resistant genes, to self-incompatible *B. rapa* species poses significant environmental risks. Therefore, it is crucial to monitor natural gene flow between GM *B. napus* and wild *B. rapa* populations in natural habitats. Assessing the impact of gene flow can be achieved by identifying historical introgression events between crops and their wild relatives through genetic diversity analyses of weedy populations, and by examining potential changes in the life cycle and behavior of introgressed populations. However, this approach presents significant challenges, as many molecular markers are shared between these species, making it difficult to differentiate between genes inherited from a common ancestor and those introduced via gene flow [14]. In order to effectively manage gene flow, models have now been developed for volunteer plants [15] and the progeny of interspecific hybrids [16] that simulate a range of scenarios, including crop rotation, roadside management, herbicide application, and conventional weed control practices. These models can provide recommendations for agronomic strategies aimed at delaying or minimizing the spread of transgenes. In the case of *Brassica rapa*, herbicide-resistant hybrid progeny could rapidly infest fields, posing a significant challenge to the use of herbicides for controlling other weed species.

In our previous study, we generated interspecific hybrids between *B. napus* and six different subspecies of *B. rapa*, followed by analysis of F1 and F2 generations to assess transgene flow [17]. The main objectives of the present study are as follows: (1) to perform interspecific hybridization between *B. napus* and *B. rapa* ssp. *pekinensis* followed by backcrossing, (2) to evaluate the crossability index between parental and backcross progenies, (3) to conduct morphological analyses, (4) to perform cytological analyses, and (5) to analyze gene flow using SSR marker analyses.

## 2. Results

### 2.1. Hybridization Potential and Crossability of B. rapa with GM B. napus

The cross-compatibility of parental lines and interspecific hybridization between *B. rapa* (♀) and GM *B. napus* (♂) lines (four lines) were initially assessed to produce F1 generations. The crossability index for parental lines was notably high, averaging at (21.5 ± 4.5), while *B. rapa* ssp. *pekinensis* exhibited a lower crossability index of 10.4 ± 3.1. However, despite this, interspecific hybridization with four different lines of GM *B. napus* showed a range of crossability indices from 10.6 ± 5.7 to 14.7 ± 5.7 (Table 1). Among the four lines of transgenic plants, TG#39 demonstrated promising hybridization potential with a higher crossability index (14.7 ± 5.7), fewer instances of vivipary (26.9%), and empty seeds (20.9%) (Table 1). Furthermore, as it was found to possess a single copy number of the gene, the transgenic line TG#39 was selected for subsequent hybridization studies. BC1 progenies were obtained by backcrossing *B. rapa* (♀) X TG#39 F1 progenies (♂). BC2 and BC3 generations were generated through backcrossing with parental *B. rapa* (♀). In comparison to F1 progenies, the backcross progenies showed a lower crossability index, ranging from 1.6 ± 0.9 to 2.2 ± 1.4 (Table 1). The occurrence of vivipary gradually decreased, while the number of empty seeds increased as the generations progressed (Table 1).

### 2.2. Morphological Variations among the Parental and Backcross Progenies

**Figure 1:**
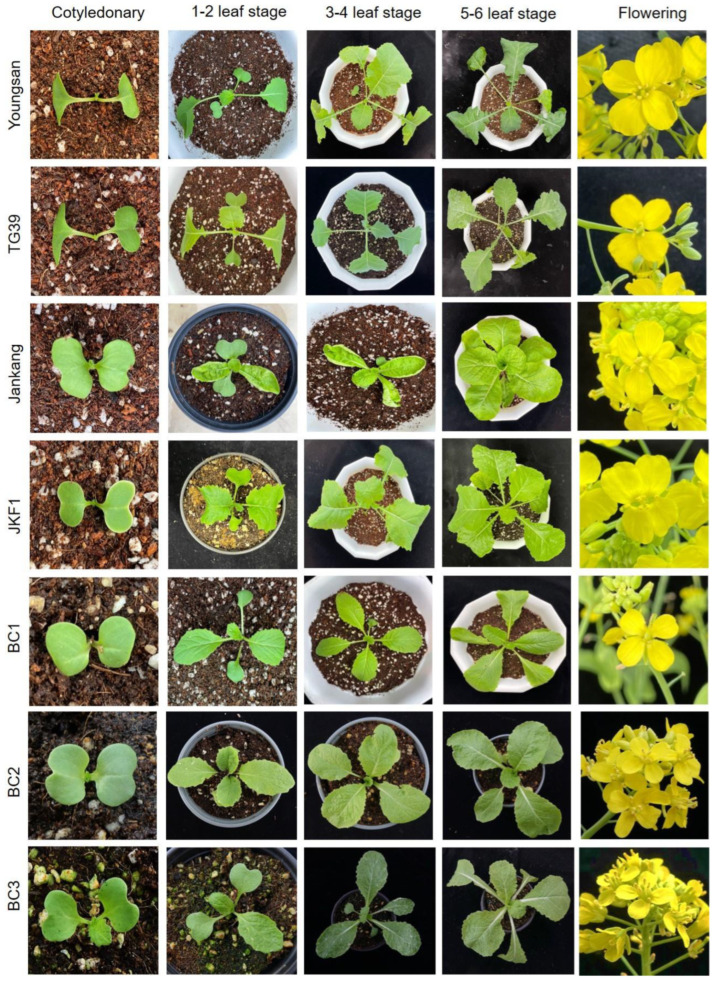
Morphological characteristics of parental, F1 hybrid, and backcross progenies in different growth stages. Representatives of *B. napus* (Youngsan), GM *B. napus* (TG39), *B. rapa* ssp. *pekinensis* (Jankang), *B. rapa* ssp. *pekinensis* F1 hybrid (JKF1), and *B. rapa* ssp. *pekinensis* backcross progenies (BC1, BC2, and BC3).

**Figure 2:**
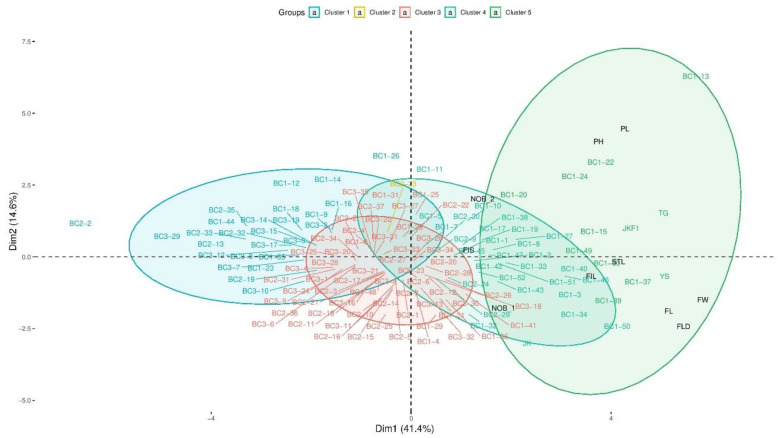
Principal coordinate analysis of parental, F1, and backcross progenies based on morphological characteristics. Representation of *B. napus* (YS), GM *B. napus* (TG), *B. rapa* ssp. *pekinensis*(JK), F1 hybrid (JKF1), and backcross progenies until BC3 (BC1, BC2, and BC3), and morphological characteristics as variables, namely plant height (PH), plant length (PL), number of branches 1 (NOB 1), number of branches 2 (NOB 2), flower length (FL), flower width (FW), flower diagonal (FL), filament length—short (FIS), filament length—long (FIL), and style length (STL).

Morphological variations in different plant growth stages, viz. seed to flowering stages of plants, are provided in Figure 1. Morphological analysis showed F1 hybrids were mostly inclined with the paternal parent *B. napus*; however, backcross progenies were found to be similar with the maternal parent *B. rapa* (Figure 1; Supplementary Table S1). Two-dimensional clustering analysis on morphological characteristics show the grouping of parental and backcross progenies (BC1, BC2, and BC3) based on changes in ten different vegetative characters (Figure 2; Supplementary Figure S1). In total, they formed four major clusters. In cluster 1, most of the backcross progenies (BC3) were grouped but not any vegetative characteristics. Clusters 2 and 3 were minor clusters with a few BC1 and BC2 progenies. The maternal parent *B. rapa* (JK), vegetative characteristics (number of branches, NOB_1 and NOB_2; filament length—short, FIS), and backcross progenies were formed as cluster 4. For most of the morphological characteristics (long style (STL), filament (FL) longer, and wider flowers (FW, FL, and FD)), F1 progenies were placed close to the paternal parent GM *B. napus* in cluster 5 (Figure 2). Two-dimensional PCA analysis showing the contribution and relativeness among morphological characteristics are shown in Supplementary Figure S2. Similarly, the morphological characteristics of BC2 progenies are found to be similar to BC1 progenies; they had a mixture of characteristics of both parents. The leaves of BC2 progenies were found to be similar to the maternal parent *B. rapa* ssp. *pekinensis* (JK) (Figure 1).

### 2.3. Cytological Analysis of Parental and Backcross Progenies

**Figure 3:**
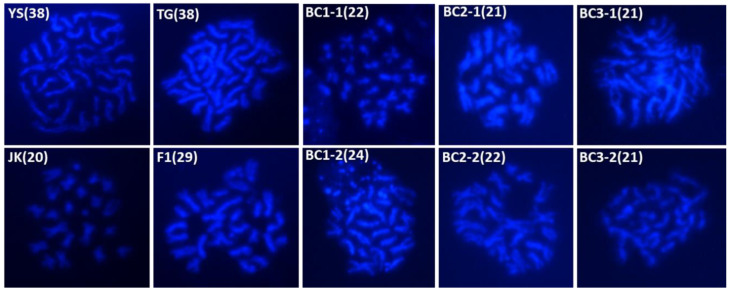
Chromosome enumeration of non-GM *B. napus* (YS), GM *B. napus* (TG), *B. rapa* (JK), and a cross combination of *B. rapa* ssp. *pekinensis* and GM *B. napus* F1 hybrid (F1) and their backcross progenies (BC1, BC2, and BC3).

In our study, we observed variable chromosome numbers in the parental genotypes, and F1 hybrid and backcross (BC1, BC2, and BC3) progenies under a fluorescence microscope (Figure 3). Chromosome counts for the parental lines were as follows: GM *B. napus* (2n = 38), and non-GM *B. napus* Youngsan (YS) (2n = 38) and *B. rapa* ssp. *pekinensis* (2n = 20) that served as controls. The F1 hybrid between *B. rapa* ssp. *pekinensis* (n = 10) and GM *B. napus* (n = 19) exhibited a chromosome number of 2n = 29 (AAC; n+n). These F1 hybrids (AAC) were used to produce backcross progenies (BC1) by crossing with parental *B. rapa* ssp. *pekinensis*. BC1 progenies displayed chromosome numbers ranging from 2n = 21 to 2n = 25. In BC2 progenies, chromosome counts varied between 2n = 21 and 2n = 22, while in BC3 progenies, they ranged from 2n = 20 to 2n = 22. Interestingly, BC2 and BC3 selfing progenies predominantly showed a specific chromosome number, with 2n = 21 being the most common in BC2 and 2n = 21 and 22 in BC3. The most frequent chromosome numbers in the backcross progenies of BC2 and BC3 were 21 and 22, respectively.

### 2.4. Molecular Marker Analyses

**Figure 4:**
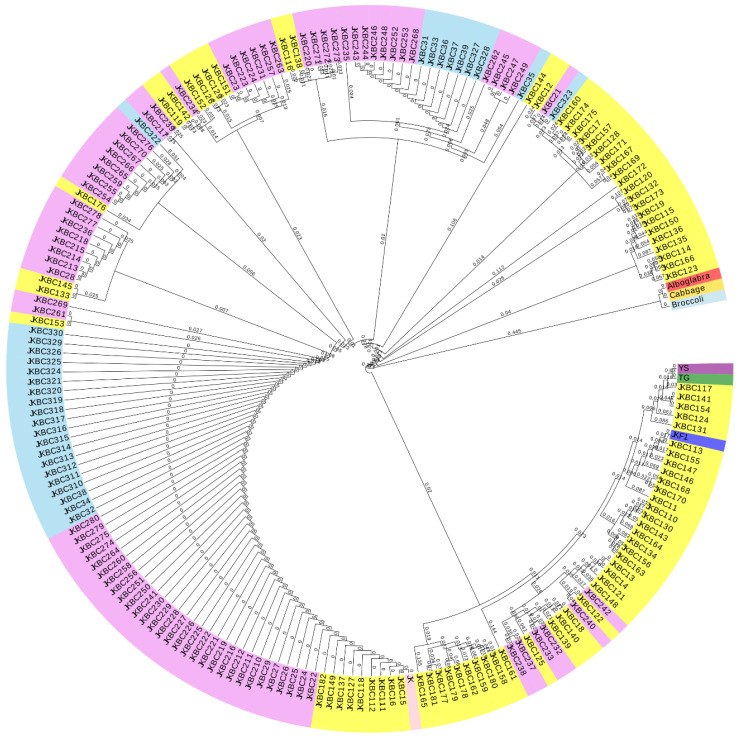
Genetic distance between parental and backcross (BC1, BC2, and BC3) progenies. The dendrogram was developed using an UPGMA clustering method based on Jaccard’s Similarity Coefficient.

Overall parental lines, F1, 82 BC1, 80 BC2, and 31 BC3 were assessed with 17 C-genome-specific SSR markers for genetic diversity analysis. BC1 progenies were mostly heterozygous and with C-genome-specific bands, whereas heterozygosity reduced when the backcross generation progressed (Table S2). As shown in Supplementary Tables S2 and S3, BC2 progenies were found to have fewer heterozygous regions and no C-genome-specific bands compared to BC1 progenies (Tables S2 and S3). However, BC3 progenies showed even fewer heterozygous bands (Table S4). An UPGMA dendrogram using Jaccard’s similarity matrix was used to evaluate the grouping of parental and backcross progenies up to BC3 generations (Figure 4). All the parental progenies and backcross progenies were grouped in three major clusters and five small clusters. Parental lines, and F1 and BC1 progenies were grouped in a single major cluster, whereas BC2 and BC3 progenies were grouped in a separate cluster. These results concurred with data of the heterozygous nature of the banding pattern using SSR markers.

## 3. Discussion

In this study, we successfully transferred a herbicide resistance gene from tetraploid *B. napus* into diploid *B. rapa* ssp. *pekinensis* (Jankang) through interspecific hybridization, followed by three generations of backcrossing. Previously, we created hybrids between *B. napus* and six different *B. rapa* subspecies, and while F1 and F2 generations were produced, they failed to generate viable subsequent generations [17]. Backcrossing F1 generations with parental lines also did not yield viable BC2 and BC3 progenies, except for *B. rapa* ssp. *pekinensis*.

Herbicide resistance genes like PAT and BAR have been incorporated into various crops to provide resistance against herbicides such as glyphosate and glufosinate [8]. The BAR gene is commonly used as a selectable marker in transgenic studies. Commercially significant GM *B. napus* was transformed with the BAR gene and other herbicide resistance genes decades ago. The risk of gene flow from *B. napus* to wild relatives is relatively low, though some studies have shown gene flow potential and volunteer formation over different periods [10,18,19]. The successful transfer of genes through pollen largely depends on the synchronization of flowering periods between herbicide-resistant (HR) crops and their wild relatives. While experimental evidence indicates that wild populations tend to have longer flowering periods compared to cultivated crops, which increases the likelihood of overlap [20], there are instances where gene flow between HR crops and their relatives is hindered due to either non-overlapping flowering periods or only brief periods of overlap.

Our research focused on introgression of the BAR gene into *B. rapa* ssp. *pekinensis* in up to three backcross generations. We observed a decreasing crossability index in backcross progenies, which may help prevent the formation of weedy *B. napus* volunteers. In the absence of direct evidence, our hypothesis suggests that factors such as pollen viability, rejection mechanisms, or pre-zygotic barriers during self-pollination may influence crossability. These factors could potentially impede pollen hydration, germination, or pollen tube growth on the stigma [21,22]. Even in cases where pollen germination and fertilization are successful, premature or viviparous germination might occur, as observed in previous studies [10,17,18,23]. In a study by Snow et al. [18], no fitness costs were observed when a glufosinate-resistant transgene was introgressed from oilseed rape into *Brassica rapa*. However, in work by Song et al. [24], reduced seed production was noted in the backcross progeny of *B. napus* × *B. juncea* crosses containing the same transgene. This variation in fitness effects is likely attributable to differences in the genomic location of the transgene. F1 hybrids from crosses between *B. napus* and its wild relatives often exhibit morphology closely resembling the maternal parent [25]. In triploid hybrids from diploid *B. rapa* and *B. napus*, Warwick et al. [26] noted that all hybrids displayed traits similar to the maternal plant, *B. rapa*. However, other studies, like those by Jørgensen and Andersen [27], observed hybrids with intermediate or paternal characteristics.

In our study, F1 hybrids primarily showed traits of the paternal parent, *B. napus*, while backcross progenies resembled the maternal parent, *B. rapa*. Notably, in BC3, three progenies had 2n = 20 chromosomes, which stabilized over successive generations, indicating that BC3 plants were fully integrated into the *B. rapa* ssp. *pekinensis* genome. Our research demonstrated the elimination of ‘C’-genome-specific genomic regions from *B. napus* through backcrossing and stabilizing the chromosome count at 20 in BC3 progenies. SSR analysis revealed that approximately 8% of *B. napus* genetic markers persisted in BC3 lines. Despite losing most *B. napus* chromosomes, we retained the genetic material conferring herbicide resistance in the advanced backcross population, which still exhibited some *B. napus* characteristics.

In previous studies, we examined gene flow involving an early flowering gene (*BrAGL20*) in F1 hybrids derived from crosses between *B. rapa* ssp. *pekinensis* and genetically modified *B. napus* [18]. Additionally, we conducted interspecific hybridization experiments involving six different subspecies of *B. rapa* and genetically modified *B. napus*, analyzing the progenies of F1, F2, and BC1 generations [17]. Diverse *B. rapa* subspecies are recognized for their substantial phenotypic and genetic variability, exhibiting varying degrees of self-incompatibility and cross-compatibility [28,29].

SSR marker analysis revealed a reduction in C-genome-specific alleles in backcross progenies. BC1 progenies displayed a higher percentage of C alleles and heterogeneous banding patterns, but this decreased progressively with each generation. Despite the extensive elimination of C-genome-specific genomic regions, the glyphosate resistance gene from the male parent *B. napus* was introgressed into BC3 progenies, suggesting that the glyphosate resistance gene located and introgressed in A-chromosome/genome regions of the Brassica plants. By the BC3 generation, C-genomic alleles were completely eliminated in the hybrids. These findings align with our cytogenetic analyses and chromosome enumeration results. Homologous recombination among the genomic regions and the chromosomes results in chromosome deletions, rearrangements, and duplications [30]. This suggests that non-random elimination of homologous sequences from diverse parental lines [31] might occur during distant hybridization in Brassica species [32]. Further research is needed to uncover the mechanisms behind chromosome elimination in Brassica distant hybridization. Significant knowledge gaps remain, particularly in understanding the impact of genetic variability on successful hybridization and fertility, the relationship between chromosome number and fertility, and the influence of selection pressure and environmental variability on hybrid performance and transgene persistence. Additionally, more research is needed to characterize population demography and the spread of hybrids within plant communities [14].

## 4. Materials and Methods

### 4.1. Plant Materials and Hybridization

Genetically modified *B. napus* ‘Youngsan’ (YS) (TG#39) incorporated the CAMV 35S-regulated bar gene and BrAGL20, which is an early flowering gene, [33]. In contrast, *B. rapa* L. ssp. *pekinensis* ‘Jangkang’ (JK) seeds were sourced from the National Agrobiodiversity Center in Jeonju, South Korea [18]. Each plant was cultivated in a 21.5 cm diameter pot using commercially available horticultural soil mix. The pots were placed 10 cm apart and received daily watering until the flowering period concluded. The plants were kept under controlled environmental conditions, with a stable temperature of 25 ± 3 °C during both the day and night. All experimental activities were performed within the biosafety greenhouse at the National Institute of Agricultural Sciences in Jeonju, South Korea.

Hybridization experiments were conducted with *B. rapa* ssp. *pekinensis* serving as the female parent (♀) and genetically modified *B. napus* as the male parent (♂). The protocols for creating the F1 generation and maintaining the plants adhered to the methods detailed in our previous study [17]. F1 hybrids (used as pollen donors) were crossed with *B. rapa* (used as the seed parent) to produce BC1 progenies. The crossability index was calculated by determining the number of seeds per pod. Herbicide resistance rates were evaluated by measuring the survival percentage of plants following herbicide treatment. Young plants were treated with 0.3% Basta (Bayer Crop Science GmbH, Manheim am Rhein, Germany) at the 4–5 leaf stage, with a second application administered four days later. For the detection of bar proteins in the backcross generation of transgenic plants, a qualitative assessment was performed on leaf tissues using a commercially available immunostrip specific to bar proteins (Agrastrip^®^ seed & leaf TraitCheck LL, Romer Labs, Newark, USA). The procedure followed the manufacturer’s guidelines for the detection assay (Supplementary Figure S3).

### 4.2. Morphological Characteristics

Morphological traits of the parental lines, F1 hybrids, and subsequent BC1 progenies were examined. These traits were classified according to the standardized descriptors for Brassica provided by the International Union for the Protection of New Varieties of Plants [34]. Vegetative characteristics included plant height (PH), panicle length (PL), and the number of branches (NOB, categorized as 1, 2, or 3). Generative traits encompassed the number of pollinated flowers (NPF), number of pods (NOP), pod-setting ratio (PSR), number of seeds (NOS), seeds per pod (SPP), vivipary (VV), non-filled seeds (NFS), flower length (FL), flower width (FW), flower diagonal (FLD), short filament length (FIS), long filament length (FIL), and style length (STL).

### 4.3. Cytological Observations

To determine chromosome numbers, we followed modified protocols based on Tagashira et al. [35] and Hoshi [36]. Root tips, harvested at 8 A.M. to coincide with peak mitotic activity, were first pre-treated with 8-hydroxyquinoline for 4 h at room temperature. Subsequently, the tips were rinsed with distilled water and fixed with 3:1 ethanol and acetic acid solution for 24 h at RT to preserve their structure. After fixation, the roots were washed again in distilled water and kept at −20 °C until further use by storing them with 70% ethanol. When ready, roots were rinsed once more in distilled H_2_O, and the meristematic regions of root tips were separated. These sections were then subjected to enzymatic treatment for one hour at 37 °C using an enzyme buffer composed of cytohelicase, cellulose, and pectolyase (each 250 mg dissolved in 25 mL of 0.01 M citrate). Post-enzyme treatment, the roots were gently crushed and cleaned with a drop of 60% acetic acid, followed by heating at 46 °C for 2 min. Later, Vectashield (H-1000) (Newark, NJ, USA) containing DAPI (4,6-diamidino-2-phenylindole) was used to stain the samples; the slides were covered with immersion oil to prevent fluorescence during examination. Chromosome counts and frequencies in parental lines, hybrids, and backcross progenies (BC1–BC3) were observed and recorded using a Nikon Eclipse 50i fluorescence microscope (Nikon, Tokyo, Japan) at 100× magnification.

### 4.4. SSR Marker Analysis

In our research, we employed seventeen SSR (simple sequence repeat) markers specific to the C genome. The details and primer sequences for these markers were previously published [17]. Young leaves were used for the extraction of genomic DNA by the CTAB (cetyl trimethyl ammonium bromide) method [37]. For PCR, a 20 µL reaction mixture was prepared, consisting of 1 µL each of forward and reverse primers (10 picomoles each), 1 µL of genomic DNA, Taq PCR mix obtained from http://cells-safe.com/ (accessed on 18 March 2023), and 18 µL of RNAase-free water. PCR amplification using a Biometra Thermal Cycler (Jena, Germany) with the conditions mentioned in our previous study [18] was carried out. The amplicons were visualized using a Qsep400 multi-channel Bio-fragment analyzer (New Taipei City, Taiwan) and a high-throughput gel electrophoresis system. We scored the presence or absence of bands in the amplification results, treating them as binary characters (H: 1,1; A: 1,0; C: 0,1). To assess the genetic relationships among the offspring, we constructed a Jaccard’s distance matrix using DARwin software version 6.0.021 for Windows [38] and performed clustering using the UPGMA method. The resulting phylogenetic tree was annotated and exported graphically using Evolview (https://evolgenius.info/evolview-v2; accessed on 21 March 2024) [39].

## 5. Conclusions

A herbicide resistance gene was successfully introduced into BC3 progenies, and herbicide resistance was observed in a cross between *B. rapa* ssp. *pekinensis* and GM *B. napus*. These genetic and phenotypic traits can be used to identify interspecific hybrids between *B. rapa* and *B. napus* early on, enabling effective prevention and monitoring of unintended introgression at the field level. Chromosome enumeration showed that, through backcrossing, the chromosome number decreased, becoming more similar to that of the maternal plants. SSR marker analysis supported these findings by showing an increase in A-genome-specific bands and a reduction in C/H bands. Overall, the study successfully introgressed the glyphosate resistance gene into BC3 lines, producing viable progenies.

## Figures and Tables

**Table 1:** Cross-compatibility of parental, interspecific F1, and backcross progenies between *B. rapa* ssp. *pekinensis* and GM *B. napus.*.

Cross Combination	Progeny	No. of Pollinated Flowers	No. of Pods	Pod-Setting Ratio (%)	Total No. of Seeds	Vivipary (%)	Empty Seeds (%)	Crossability Index (No. of Seeds/Pods)
*B. napus*	Parental	410	282	68.8	6073	89 (1.47)	249 (4)	21.5 ± 4.5
*B. rapa* ssp. *pekinensis*	Parental	268	159	59.4	1657	12 (0.72)	454 (27)	10.4 ± 3.1
*B. rapa* ssp. *pekinensis* (♀) X GM *B. napus* (TG#19) (♂)	F1 hybrid	123	61	49.5	480	152 (68.2)	54 (24.2)	11.2 ± 1.3
*B. rapa* ssp. *pekinensis* (♀) X GM *B. napus* (TG#39) (♂)	F1 hybrid	1282	540	42.1	1926	518 (26.9)	403 (20.9)	14.7 ± 5.7
*B. rapa* ssp. *pekinensis* (♀) X GM *B. napus* (TG#53) (♂)	F1 hybrid	252	115	45.6	531	350 (65.9)	146 (27.5)	10.6 ± 5.7
*B. rapa* ssp. *pekinensis* (♀) X GM *B. napus* (TG#74) (♂)	F1 hybrid	337	206	61.1	433	240 (55.4)	131 (30.3)	14.4 ± 2.6
*B. rapa* ssp. *pekinensis* (♀) X F1 hybrid	BC1 hybrid	1640	355	21.6	781	218 (27.9)	210 (26.9)	2.2 ± 1.4
*B. rapa* ssp. *pekinensis* (♀) X BC1 hybrid (♂)	BC2 hybrid	2992	351	11.7	946	100 (10.6)	426 (45.0)	2.7 ± 1.9
*B. rapa* ssp. *pekinensis* (♀) X BC2 hybrid (♂)	BC3 hybrid	595	46	7.7	74	5 (6.8)	32 (43.2)	1.6 ± 0.9

## Data Availability

The original contribution presented in the study is publicly available.

## Supplementary Materials

The following supporting information can be downloaded at https://www.mdpi.com/article/10.3390/plants13202863/s1: Figure S1. Cluster representation of B. napus (YS), GM B. napus (TG), B. rapa ssp. pekinensis, B. rapa F1 hybrid, and B. rapa ssp. pekinensis (BC1) with 10 agricultural characteristics; Figure S2. Two-dimensional PCA showing the contribution and relationships among ten morphological characteristics; Figure S3. Immunostrip specific to bar protein results for backcross progenies. A—BC1 progenies, B—BC2 progenies, C—BC3 progenies; Table S1. Ten different morphological differences among parental, F1 hybrid, and backcross progenies (BC1, BC2, and BC3); Table S2. Chromosome segregation analysis of BC1 progenies using C-genome-specific SSR markers; Table S3. Chromosome segregation analysis of BC2 progenies using C-genome-specific SSR markers; Table S4. Chromosome segregation analysis of BC3 progenies using C-genome-specific SSR markers.
