# Supplementary material for: Introgression of Herbicide-Resistant Gene from Genetically Modified Brassica napus L. to Brassica rapa through Backcrossing
Source: Plants (Basel). 2024 Oct 13;13(20):2863. doi: 10.3390/plants13202863 (PMC11510986; doi:10.3390/plants13202863)
Supplement: Supplementary file 1 [file plants-13-02863-s001.zip › plants-3160197-supplementary/3 Supplementary Figures.pptx]

## Slide 1
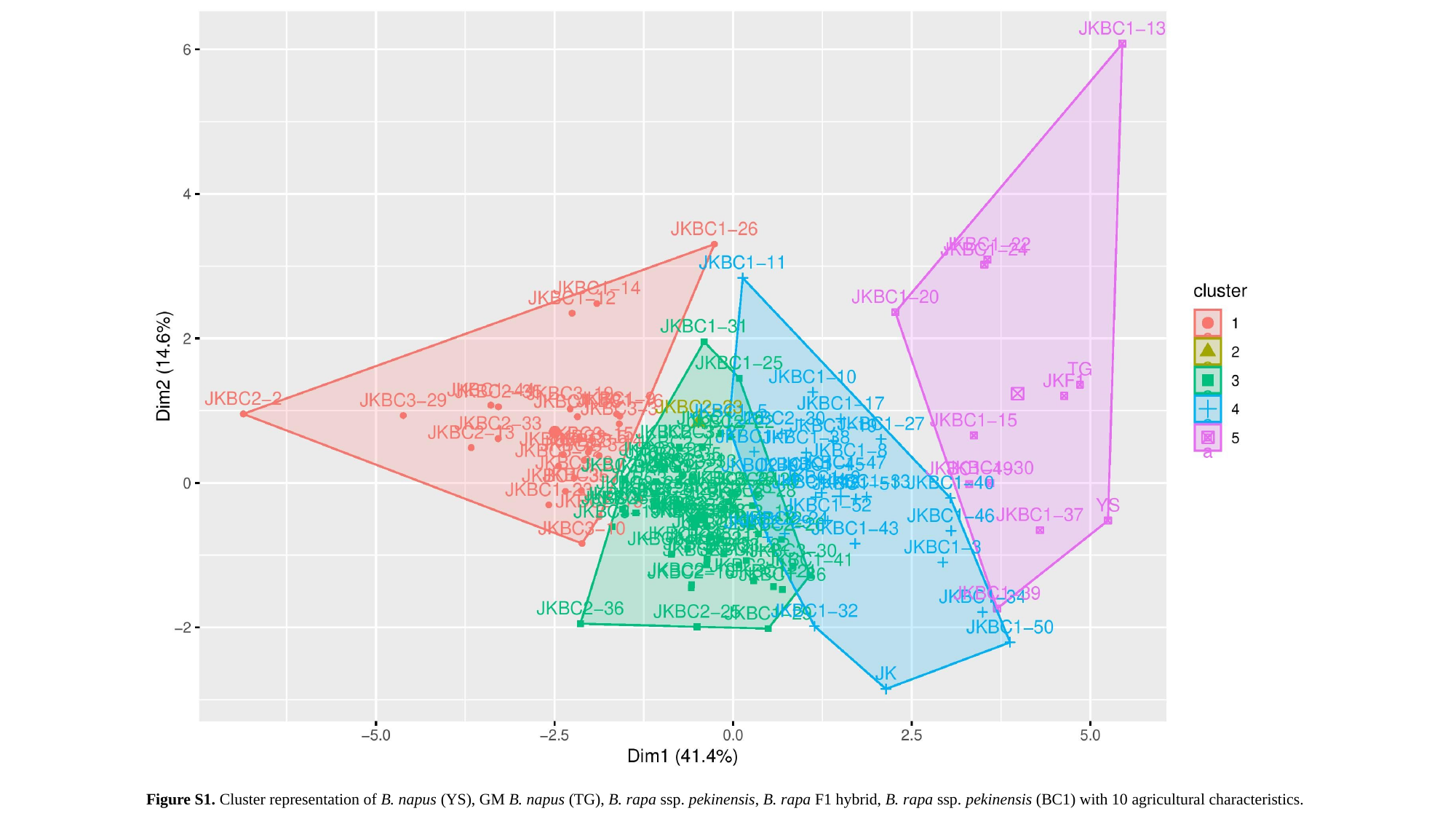

Figure S1. Cluster representation of B. napus (YS), GM B. napus (TG), B. rapa ssp. pekinensis, B. rapa F1 hybrid, B. rapa ssp. pekinensis (BC1) with 10 agricultural characteristics.

## Slide 2
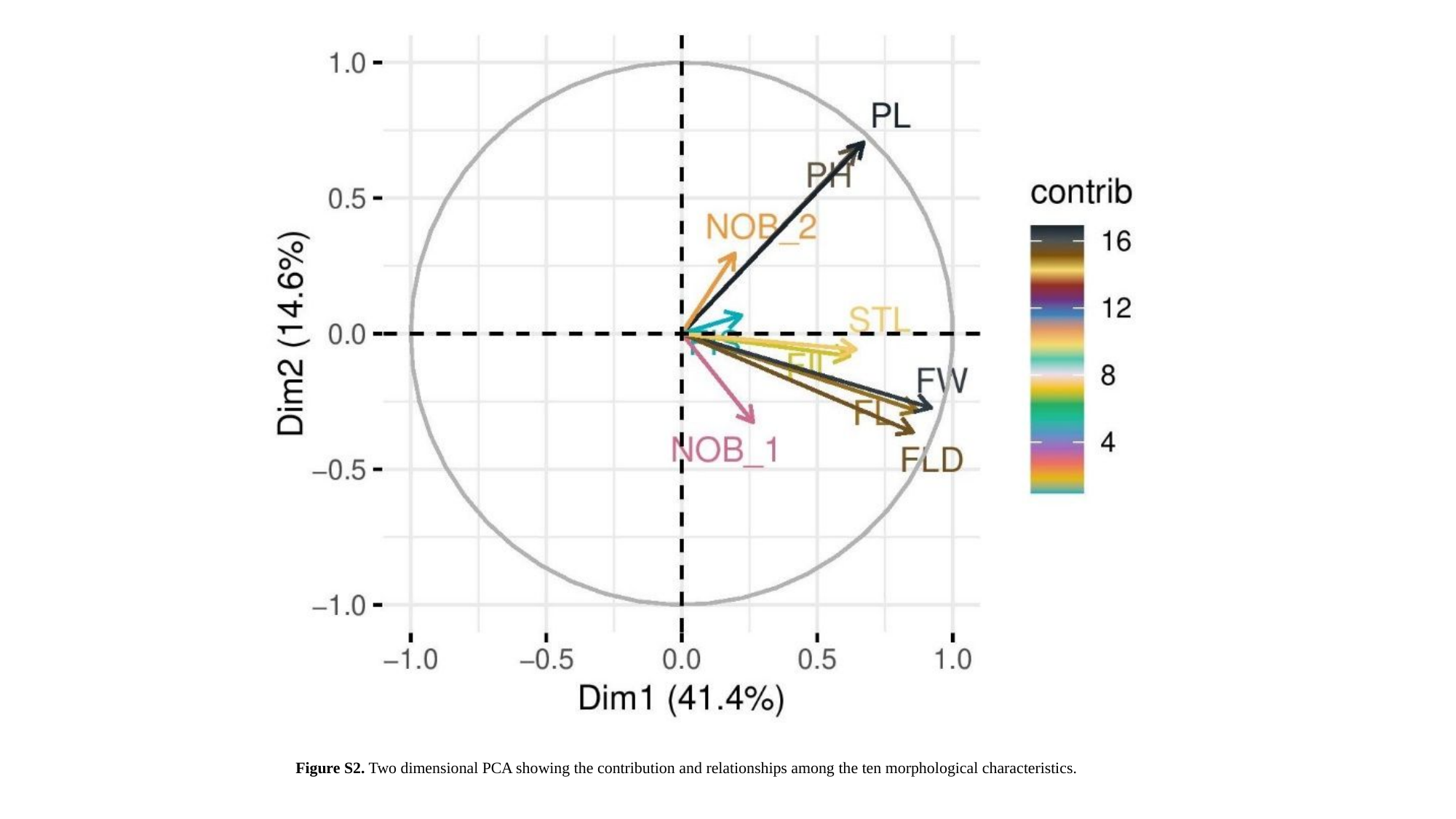

Figure S2. Two dimensional PCA showing the contribution and relationships among the ten morphological characteristics.

## Slide 3
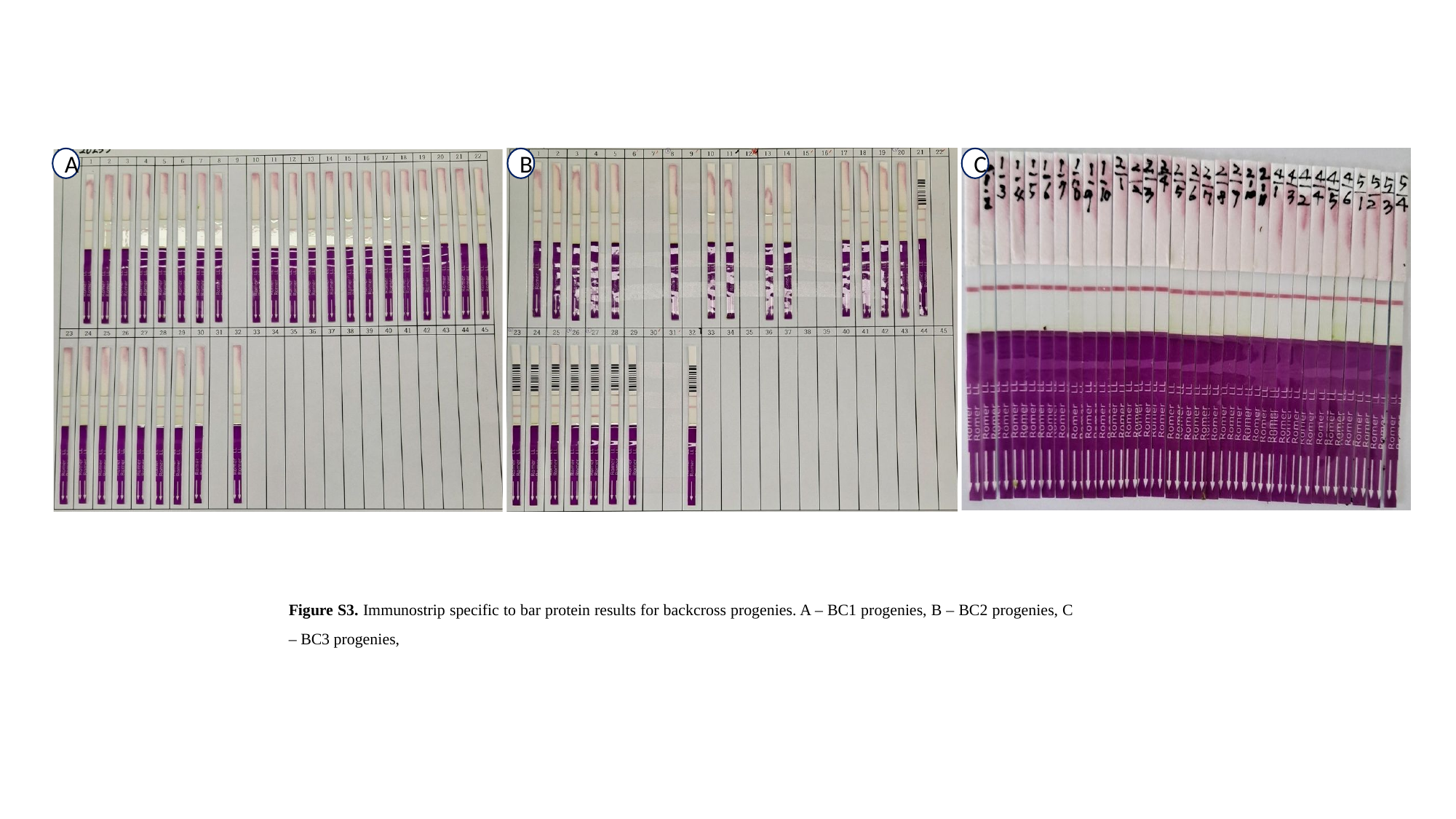

A
B
C
Figure S3. Immunostrip specific to bar protein results for backcross progenies. A – BC1 progenies, B – BC2 progenies, C – BC3 progenies,
